# Polydeoxyribonucleotide (PDRN) Selectively Promotes Osteoblast Differentiation Without Affecting Osteoclastogenesis

**DOI:** 10.3390/md24030100

**Published:** 2026-03-03

**Authors:** Younghoon Jeon, Eunjung Heo, Xian Jin, Dong-Kyo Lee, Xiangguo Che, Hyun-Ju Kim, Sung-Hye Byun, Je-Yong Choi, Jeongkyu Choi, Jinyoung Oh

**Affiliations:** 1 Department of Anesthesiology and Pain Medicine, School of Medicine Kyungpook National University, Daegu 41944, Republic of Korea; 2 Department of Anesthesiology and Pain Medicine, Kyungpook National University Hospital, Daegu 41944, Republic of Korea; 3 Department of Biochemistry and Cell Biology, Cell and Matrix Research Institute, School of Medicine, Kyungpook National University, Daegu 41944, Republic of Korea; 4 Department of Anesthesiology and Pain Medicine, Kyungpook National University Chilgok Hospital, Daegu 41404, Republic of Korea

**Keywords:** polydeoxyribonucleotide, osteoblast, differentiation, adenosine A_2A_ receptor, bone regeneration, osteoclast

## Abstract

Developing novel anabolic agents for bone regeneration remains a clinical priority. Polydeoxyribonucleotide (PDRN) exhibits tissue-regenerative properties, but its direct cellular effects on bone remodeling remain unclear. This in vitro study investigated PDRN’s effects on osteoblast (MC3T3-E1) and osteoclast (primary bone marrow-derived macrophages) differentiation. We evaluated metabolic activity, gene/protein expression, and specific differentiation markers using MTS, qRT-PCR, Western blotting, and functional assays (ALP, Alizarin Red S, TRAP, pit formation). In osteoblasts, PDRN dose-dependently modulated metabolic activity while upregulating the early transcription factor Runx2. PDRN significantly enhanced osteoblast differentiation, evidenced by increased ALP activity, elevated mineralized matrix deposition, and robust upregulation of osteocalcin and Runx2. Conversely, PDRN exhibited no direct effect on osteoclast precursor metabolic activity, differentiation, or resorptive function. These findings support a working hypothesis in which PDRN selectively promotes osteoblast differentiation without directly affecting osteoclastogenesis. While further pharmacological investigations are required to definitively elucidate the specific purinergic receptor mechanisms, our results highlight PDRN as a promising candidate anabolic agent for bone regeneration.

## 1. Introduction

Skeletal homeostasis is maintained through a finely regulated balance between the activities of bone-forming osteoblasts and bone-resorbing osteoclasts. Metabolic bone diseases such as osteoporosis arise when osteoclastic resorption surpasses osteoblastic formation, leading to reduced bone mass and microarchitectural deterioration, thereby increasing fracture risk [1]. Accordingly, therapeutic strategies for metabolic bone diseases such as osteoporosis aim either to inhibit osteoclast function or to enhance osteoblast activity or simultaneously modulate both to restore skeletal balance [2,3].

Adenosine signaling acts as a pivotal regulator of bone homeostasis, governed by a tightly controlled system of extracellular nucleotide metabolism and transport. Under physiological conditions, extracellular adenosine levels are tightly regulated by a dynamic balance between nucleotide release, enzymatic metabolism, and cellular uptake [4]. In the bone microenvironment, intracellular adenosine triphosphate (ATP) is actively released into the extracellular space via regulated vesicular exocytosis or conductive channels, such as connexin 43 hemichannels and pannexin 1 channels, often in response to mechanical stimuli [5]. Once released, nucleotides undergo a sequential enzymatic cascade. ATP and adenosine diphosphate (ADP) are hydrolyzed to adenosine monophosphate (AMP) by ectonucleoside triphosphate diphosphohydrolase-1 (CD39) [6]. Crucially, tissue-nonspecific alkaline phosphatase (TNAP), a key osteoblast marker, also hydrolyzes ATP and pyrophosphate (PPi), thereby regulating mineralization and providing a substrate for adenosine generation [7,8]. Subsequently, AMP is rapidly converted into adenosine by ecto-5′-nucleotidase (CD73) [6]. Once generated, adenosine exerts its physiological effects by binding to four distinct G-protein-coupled receptors (GPCRs): the adenosine A_1_, A_2A_, A_2B_, and A_3_ receptors [9,10]. Signaling is terminated by the deamination of adenosine to inosine via adenosine deaminase (ADA) or by its bidirectional re-uptake into cells through equilibrative nucleoside transporters (ENTs) (predominantly ENT1 and ENT2), which strictly regulate local adenosine concentrations based on gradients [11]. Intracellularly, adenosine enters the purine salvage pathway, where it is re-phosphorylated to AMP by adenosine kinase (ADK) to support nucleic acid synthesis [12].

Among the adenosine receptors, the adenosine A_2A_ receptor has been identified as a key driver of osteoblast differentiation and bone formation [6,13]. A_2A_ receptor is expressed on both osteoblasts and osteoclasts; its activation in osteoblasts promotes bone formation through intracellular signaling pathways, whereas in osteoclasts, it has been shown to suppress differentiation [14,15,16,17,18]. These dual effects position A_2A_ receptor as a promising therapeutic target for maintaining skeletal integrity.

Polydeoxyribonucleotide (PDRN), a mixture of deoxyribonucleotides extracted from salmon sperm DNA, has emerged as a regenerative agent with anti-inflammatory and tissue-reparative properties [19]. PDRN activates the adenosine A_2A_ receptor, promoting angiogenesis, wound healing, and cellular proliferation, and anti-inflammatory effects in various tissue contexts [19,20]. Previous in vitro and in vivo studies have demonstrated its potential in bone healing, particularly in models of bone defect [21]. PDRN has been reported to stimulate osteoblast proliferation and differentiation, supporting its candidacy as a potent anabolic agent for skeletal repair [13,22].

Despite growing evidence of its osteogenic potential, the precise effects of PDRN on osteoclast differentiation and function remain poorly understood. Given that bone remodeling depends on a tightly regulated interplay between bone formation and resorption, a comprehensive evaluation of PDRN’s impact on both osteoblasts and osteoclasts is essential. Although several studies suggest that PDRN-loaded scaffolds may suppress RANKL-induced osteoclastogenesis in complex in vivo or scaffold-based environments, the direct cellular effects of PDRN alone under controlled conditions remain to be clarified [22,23].

In this study, we investigated the direct in vitro effects of PDRN on osteoblast and osteoclast differentiation. MC3T3-E1 pre-osteoblasts and primary bone marrow-derived macrophages (BMMs) were employed to assess osteogenic and osteoclastic responses, respectively. Osteoblast differentiation was evaluated via alkaline phosphatase (ALP) activity, mineralization assays, and expression of osteogenic markers. In parallel, osteoclastogenesis was assessed using tartrate-resistant acid phosphatase (TRAP) staining, resorptive pit formation, and analysis of osteoclast differentiation markers. These experiments aimed to elucidate the cellular actions of PDRN on bone remodeling and to explore its therapeutic potential as a novel agent for bone regeneration and osteoporosis management.

## 2. Results

### 2.1. PDRN Upregulates Early Osteogenic Markers Concomitant with Reduced Cell Metabolic Activity and Cell Number

**Figure 1:**
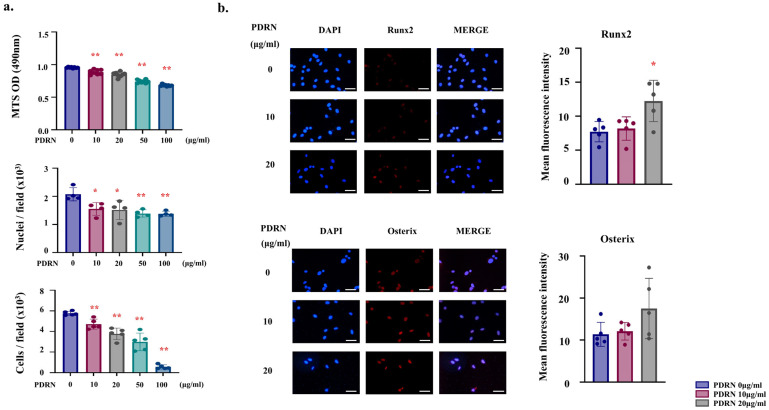
PDRN modulates metabolic activity and promotes early osteogenic differentiation in MC3T3-E1 cells. (**a**) Effects of PDRN on MC3T3-E1 metabolic activity and cell number at 24 h. Cell metabolic activity was assessed by MTS assay (upper), and cell number was quantified by direct nuclei and cell counting per field (middle and lower). Note that PDRN treatment resulted in a dose-dependent reduction in both metabolic activity and cell number. Individual dots represent independent biological replicates (*n* = 5). (**b**) Immunofluorescence staining of early osteogenic markers Runx2 (upper) and Osterix (lower) at 24 h. Nuclei were counterstained with DAPI (blue). Scale bar, 50 μm. Quantitative analysis of fluorescence intensity (right) shows significant upregulation of Runx2 and Osterix in the 20 µg/mL PDRN-treated group. For quantification, four systematically selected microscopic fields (four cardinal points) were analyzed from each of five independent biological replicates (*n* = 5). Each data point represents mean fluorescence intensity of one biological replicate. Data are presented as mean ± SD. Statistical significance was determined by one-way ANOVA followed by Tukey’s post hoc test. * *p* < 0.05, ** *p* < 0.01 vs. PDRN 0 µg/mL. Blue bars represent PDRN 0 µg/mL (control), red bars represent PDRN 10 µg/mL, and green bars represent PDRN 20 µg/mL.

To investigate the initial cellular response to PDRN, we first assessed the cell metabolic activity and cell number in MC3T3-E1 pre-osteoblast after 24 h of treatment. The MTS assay revealed a significant, dose-dependent reduction in metabolic activity in the PDRN-treated groups compared to the control (Figure 1a, upper). To confirm whether this reduction reflected a true decrease in cell number, we performed direct nuclei and cell counting. Consistent with the metabolic data, the number of nuclei and cells per field was significantly lower in the PDRN-treated groups (Figure 1a, middle and lower). Concurrently, we examined the expression of early osteogenic transcription factors. Immunofluorescence analysis at 24 h demonstrated a robust upregulation of Runx2 in the nuclei of PDRN-treated cells compared to controls (Figure 1b).

To determine whether the observed reduction in metabolic activity was attributable to cytotoxicity, we performed an additional time-course assay with increased biological replicates. The results confirmed that while metabolic activity was lower in PDRN-treated groups at 24 h, it increased in all groups by 48 h, demonstrating that the cells remained viable and proliferative (Figure S1).

Based on these results and preliminary screening, PDRN concentrations of 10 and 20 μg/mL were selected for subsequent assays, as they induced the most significant osteogenic signals (e.g., Runx2 upregulation) while maintaining adequate cell metabolic activity without overt cytotoxicity. Immunofluorescence staining showed a significant increase in Runx2 expression in the PDRN-treated groups relative to controls, whereas Osterix expression did not exhibit significant changes during this early phase (Figure 1b).

### 2.2. PDRN Promotes Osteoblast Differentiation and Mineralization

**Figure 2:**
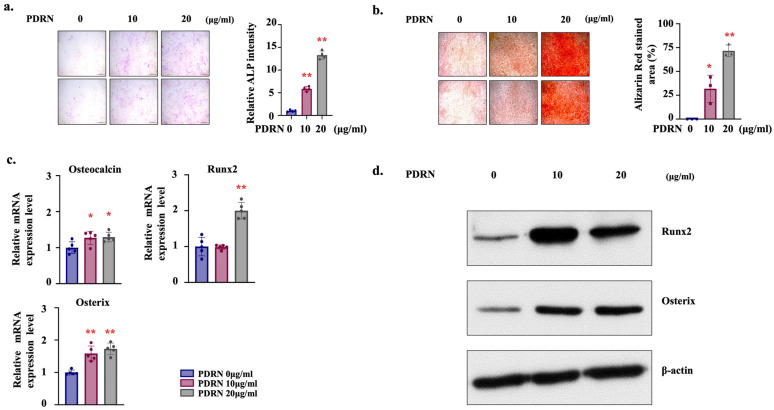
PDRN promotes osteogenic differentiation in MC3T3-E1 cells. (**a**) ALP staining (left) and quantitative analysis of ALP activity (right) at day 7 of culture. Individual dots represent independent biological replicates (*n* = 4). (**b**) Alizarin Red S staining (left) and quantitative analysis (right) at day 28 of culture. Individual dots represent independent biological replicates (*n* = 3). (**c**) mRNA expression of osteogenic markers (Osteocalcin, Runx2, and Osterix), assessed by qRT-PCR. Note that transcriptional upregulation was confirmed with high statistical power. (**d**) Protein levels of Runx2 and Osterix, assessed by Western blot. The shown images are representative Western blot images. Sample sizes vary by assay method to optimize statistical rigor. While Western blotting provides qualitative confirmation, the primary quantitative assessment is provided by the robust qRT-PCR analysis (*n* = 5) in panel (**c**). Error bars indicate SD. Statistical significance was determined by one-way ANOVA followed by Tukey’s post hoc test. * *p* < 0.05, ** *p* < 0.01 vs. PDRN 0 µg/mL. Scale bar, 500 µm.

We next evaluated the effects of PDRN on osteoblast differentiation and mineralization in MC3T3-E1 cells using ALP staining and Alizarin Red S staining. Since ALP activity typically peaks during the early-to-mid phase of osteogenesis, we measured ALP staining at Day 7. Conversely, mineralization is a late-stage marker, which was assessed by Alizarin Red S (ARS) staining at Day 28. Treatment of MC3T3-E1 cells with 10 μg/mL and 20 μg/mL PDRN significantly enhanced ALP activity at day 7 and mineralized matrix deposition at day 28. Notably, both ALP activity and mineralization were higher in the 20 μg/mL group compared to the 10 μg/mL group (Figure 2a,b). Quantitative RT-PCR analysis revealed significant upregulation of osteogenic gene expression, including *Osteocalcin*, *Runx2*, and *Osterix*, in PDRN-treated groups, with *Runx2* showing the most pronounced increase at 20 μg/mL (Figure 2c). Consistently, Western blot analysis confirmed elevated protein levels of Runx2 and Osterix in PDRN-treated cells compared to controls (Figure 2d), further supporting the osteogenic effect of PDRN.

### 2.3. PDRN Does Not Directly Affect Osteoclast Precursor Viability or Differentiation

**Figure 3:**
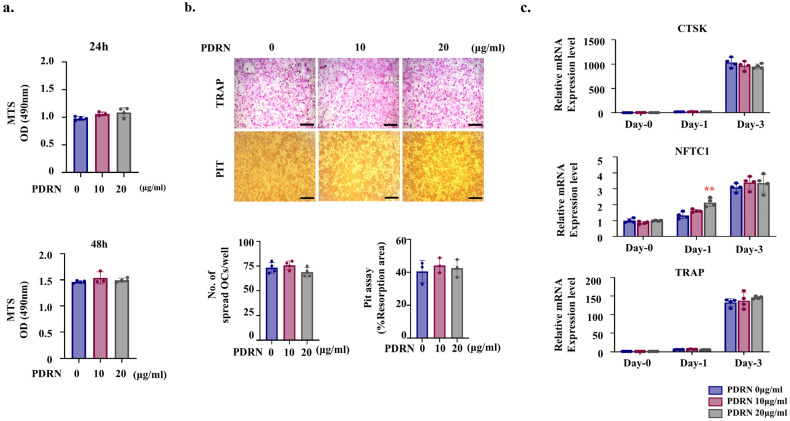
Effects of PDRN on osteoclast metabolic activity and differentiation. (**a**) MTS assay at 24 h and 48 h of culture. (**b**) TRAP staining and resorption pit assay at day 3 of culture. TRAP-positive multinucleated cells were identified as osteoclasts. (**c**) mRNA expression of osteoclast markers (CTSK, NFATc1, and TRAP), assessed by qRT-PCR. Individual data points (dots) represent independent biological replicates (*n* = 4). Data are presented as mean ± SD. Error bars indicate SD. Statistical significance was determined by one-way ANOVA followed by Tukey’s post hoc test. Scale bars, 500 µm. ** *p* < 0.01 vs. PDRN 0 µg/mL.

To assess the effects of PDRN on osteoclastogenesis, BMMs were treated with 0, 10, and 20 μg/mL PDRN. Cell metabolic activity was evaluated using the MTS assay, as performed for MC3T3-E1 pre-osteoblasts. In the MTS assay, PDRN treatment at 10 and 20 µg/mL showed no significant effect on BMM (osteoclast precursor) metabolic activity compared to the control group (Figure 3a). Osteoclast activity, assessed by TRAP staining and resorption pit assays, showed no significant differences between groups (Figure 3b). At the molecular level, although NFATc1 expression was transiently upregulated at Day 1 in the 20 µg/mL group, no significant differences were observed at Day 3. Furthermore, other key osteoclast differentiation markers, such as Cathepsin K (CTSK) and TRAP, showed no significant changes between the PDRN-treated and control groups throughout the differentiation period (Figure 3c).

## 3. Discussion

**Figure 4:**
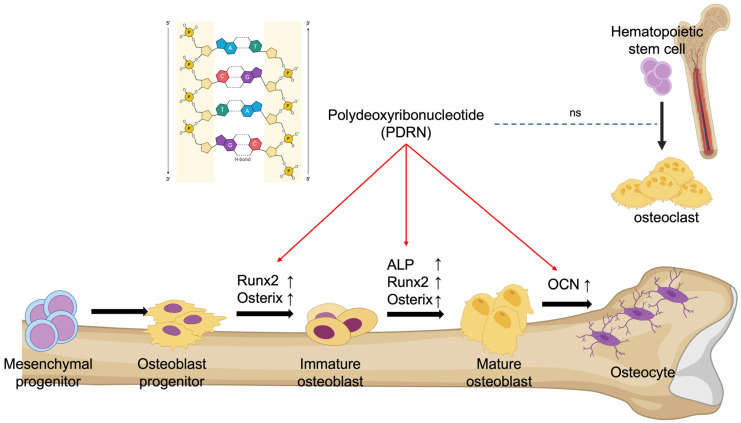
Schematic representation of experimental findings. PDRN promotes osteoblast differentiation with minimal impact on osteoclasts. Red arrows indicate the stimulatory effects of PDRN on osteoblast differentiation. Black arrows represent the sequential stages of osteoblast differentiation. The blue dashed line indicates no significant effect of PDRN on osteoclastogenesis (ns, not significant).

Therapeutic strategies for osteoporosis are broadly classified into anti-resorptive, osteoclast-targeted interventions and anabolic, osteoblast-targeted approaches. Existing anabolic agents include teriparatide, a parathyroid hormone (PTH) analogue that stimulates osteoblast activity, and romosuzumab, which promotes bone formation and inhibits bone resorption by blocking sclerostin [24]. Our findings indicate that PDRN, unlike conventional anabolic agents, directly enhances osteoblast differentiation without affecting osteoclasts (Figure 4). Specifically, PDRN enhanced biochemical enzymatic activity at the early stage (day 7) and promoted mineralized matrix deposition at the terminal stage (day 28), confirming its efficacy across the entire osteogenic progression. This suggests that systemically administered PDRN, at appropriate dosages, may offer a novel therapeutic alternative with a potentially improved safety profile compared to existing osteoporosis treatments.

A key observation in this study is the dose-dependent reduction in metabolic activity and cell number in pre-osteoblasts 24 h after PDRN treatment. Although this initial metabolic reduction could be misinterpreted as cytotoxicity, our extended time-course analysis revealed that metabolic activity increased in all groups by 48 h (Figure S1), confirming that the cells remained viable. Crucially, the reduction in cell number—confirmed by direct counting (Figure 1a)—was concomitant with a robust upregulation of Runx2 (Figure 1b). While direct cell cycle analysis (e.g., via BrdU or Ki67) was not performed, this inverse relationship—a reduction in metabolic activity and cell number coupled with the induction of differentiation markers—phenotypically resembles the early stages of the ‘proliferation-differentiation switch’ widely described in osteobiology [25,26,27]. During skeletal development, progenitor cells must exit the cell cycle and initiate lineage-specific gene expression. This shift is typically accompanied by a reduction in metabolic rate, as cellular resources are redirected from proliferation to matrix protein synthesis [28,29]. Therefore, we cautiously hypothesize that the PDRN-induced modulation may represent a metabolic shift favoring differentiation rather the cytotoxicity. We acknowledge that the MTS assay primarily measures metabolic activity, and further precise proliferation assays are required to definitively validate this transition.

This modulation of metabolic activity stands in contrast to previous studies, such as Guizzardi et al. (2003) and Kim et al. (2021) which reported proliferative effects of PDRN [13,22]. These discrepancies can be attributed to distinct experimental conditions and physiological contexts. First, regarding concentration, Guizzardi et al. (2003) utilized a significantly higher dose of PDRN (100 µg/mL) in human primary osteoblast [13]. High concentrations of nucleotides may predominantly fuel the salvage pathway for DNA synthesis, thereby driving proliferation [19]. In contrast, our study employed a lower range (10–20 µg/mL). Our results suggest that at this specific dosage, the putative A_2A_ receptor-mediated signaling effects (Runx2 upregulation) may predominate over the proliferative drive, effectively shifting the cellular balance towards osteogenic commitment. Second, regarding cell type, Kim et al. (2021) employed MG-63 cells, an osteosarcoma cell line, within 3D scaffolds [22]. Unlike the non-transformed MC3T3-E1 pre-osteoblasts used in our study, tumor-derived cells often exhibit deregulated cell cycle control and may continue to proliferate despite differentiation cues. In our model, the observed pattern of metabolic activity is consistent with the physiological requirements for differentiation, where the robust induction of Runx2 necessitates cell cycle arrest to allow for matrix synthesis and mineralization.

Mechanistically, our findings align with the existing literature, suggesting a potential functional interaction between adenosine signaling and osteogenic pathways. Specifically, previous research has established that A_2A_ receptor activation crosstalks with the canonical Wnt/β-catenin pathway. Borhani et al. (2019) demonstrated that A_2A_ receptor signaling facilitates the nuclear accumulation of β-catenin, which acts as a co-activator to drive the transcription of osteogenic genes, most notably Runx2 [16]. Our observation of robust Runx2 upregulation (Figure 2) is phenotypically consistent with this established mechanism, suggesting that PDRN likely acts through this canonical osteogenic cascade. Since Runx2 is a direct downstream target of Wnt/β-catenin signaling, its elevated expression further supports the hypothesis that PDRN successfully activated this canonical osteogenic cascade [30].

Crucially, Runx2 serves as the central mediator of this switch; high levels of Runx2 not only drive osteoblast-specific gene expression but also induce cell cycle arrest in the G1 phase by interacting with cell cycle regulators [31]. Therefore, the robust upregulation of Runx2 observed in the 20 µg/mL PDRN-treated group raises the possibility that PDRN might redirect cellular machinery from proliferation towards terminal differentiation, potentially involving pathways parallel to the A_2A_ receptor-Wnt/β-catenin axis. However, without direct pharmacological blockade, this remains a speculative hypothesis.

Thus, the observed initial reduction in metabolic activity is not a limitation but a hallmark of osteogenic activation. This interpretation resolves the apparent paradox of reduced metabolic activity coinciding with enhanced differentiation and mineralization (Figure 2). This functional trade-off is consistent with the fundamental ‘inverse relationship between proliferation and differentiation’ described in osteobiology [32,33]. Notably, a similar phenotypic shift—modulation of metabolic activity coupled with enhanced differentiation—has been reported for other osteogenic agents, such as simvastatin and intermittent PTH [34,35]. This suggests that metabolic reprogramming is a common prerequisite for robust osteogenic commitment, regardless of the distinct chemical structures or upstream signaling mechanisms of the inducing agents.

We acknowledge that the existing literature presents apparent contradictions regarding the role of adenosine signaling, which appears to be highly dependent on the differentiation stage of the cells. For instance, Costa et al. (2011) reported that in human primary bone marrow-derived mesenchymal stem cells (hBM-MSCs), selective A_2A_ receptor activation promoted cell proliferation but delayed osteogenic differentiation [36]. This suggests that in early-stage stem cells, A_2A_ receptor signaling may favor the maintenance of a proliferative pool. However, our study utilized MC3T3-E1 cells, which are committed pre-osteoblasts. In this specific cellular context, PDRN treatment robustly promoted osteogenic differentiation markers (Runx2, ALP) and mineralization. This discrepancy suggests that adenosine signaling may exert a biphasic role: supporting proliferation in multipotent progenitors while driving terminal differentiation in lineage-committed osteoblasts. Furthermore, PDRN provides a mixture of nucleotides that may activate salvage pathways, a mechanism distinct from that of the pure synthetic A_2A_ receptor agonists. This unique dual action may potentially contribute to the potent osteogenic efficacy observed in our results [19].

The dynamics of adenosine receptor expression following PDRN treatment warrant further discussion. Although we did not quantify receptor levels in this study, previous reports suggest that adenosine receptors, particularly A_2A_ receptor, may undergo internalization or desensitization, which results in apparent downregulation of the receptor, upon prolonged agonist exposure or during the late stages of differentiation [36]. However, the robust upregulation of Runx2 and the enhanced mineralization observed in our study indicate that PDRN-induced signaling remains sufficiently active to drive osteogenic commitment, irrespective of potential fluctuations in receptor surface density.

The pharmacological stability of PDRN in the culture microenvironment is an important factor in its efficacy. Although PDRN is subject to cleavage by non-specific plasma nucleases present in the serum, with a reported half-life of approximately 3 h, this degradation is functionally advantageous. PDRN acts as a macromolecular reservoir that slowly releases active deoxyribonucleotides and nucleosides, thereby providing a sustained source of ligands for adenosine receptors and substrates for the salvage pathway [19,37]. This ‘pro-drug’ like behavior likely prevents the rapid depletion of active metabolites, contributing to the prolonged osteogenic effects observed in our study.

The absence of a direct effect of PDRN on osteoclasts (Figure 3) is noteworthy. Although a transient upregulation of NFATc1 was observed at the early stage (Day 1), it did not translate into sustained expression or functional maturation, as evidenced by the lack of significant differences in TRAP staining and resorption activity. While previous studies reported that PDRN-containing scaffolds demonstrated not only enhanced bone regeneration but also potential attenuation of osteoclastogenesis, our results from a controlled monoculture system unequivocally demonstrate that PDRN does not directly inhibit osteoclast differentiation [22].

This finding stands in contrast to previous in vitro reports by Mediero et al. (2012) who demonstrated that A_2A_ receptor agonist directly suppress osteoclast formation via NF-κB inhibition [17]. This discrepancy highlights the critical pharmacological distinction between pure synthetic agonists and PDRN. Unlike potent synthetic ligands that may forcibly saturate receptors to trigger inhibitory signals, PDRN functions as a prodrug, releasing physiological levels of adenosine and nucleotides. Our data suggest that PDRN’s mode of action does not involve the direct intrinsic suppression of osteoclast precursors observed with high-affinity agonists. Instead, its reported anti-resorptive effects in tissue contexts are likely mediated indirectly, potentially through osteoblast-dependent mechanisms regulated by canonical Wnt signaling [38]. As this study focuses on the intrinsic differentiation potential of individual cell types, further co-culture investigations are warranted to fully elucidate the paracrine crosstalk between PDRN-treated osteoblasts and osteoclasts.

Taken together, these findings position PDRN as a promising anabolic agent with a distinct mechanism of action compared to existing bone formation promoters such as teriparatide or romosozumab. Importantly, PDRN has an established safety record in localized tissue regeneration, with minimal systemic side effects. This contrasts with existing osteoporosis therapies, which carry risks such as osteonecrosis of the jaw and atypical femoral fractures when administered systemically.

While these findings offer promising insights, this study has several limitations that warrant consideration. Firstly, this study was conducted in an in vitro monoculture system, which does not fully capture the complex cellular interactions present in vivo. Consequently, indirect effects mediated by osteoblast–osteoclast crosstalk could not be assessed. Secondly, a major limitation of this study is the absence of direct validation using selective pharmacological antagonists. Bone cells autonomously release ATP and adenosine, which can exert a basal ‘tonic’ activity via ectonucleotidases [5,6]. Without evaluating selective A_2A_ receptor antagonists alone and in combination with PDRN, it is premature to definitively conclude that the observed osteogenic effects are exclusively mediated by the A_2A_ receptor. Furthermore, purinergic signaling is highly complex and context-dependent; while some studies show that A_2A_ receptor activation promotes osteogenesis, others reported delayed differentiation in primary stem cells or direct osteoclast suppression [17,36]. Thus, our mechanistic discussion remains a hypothesis providing a phenotypic basis for future pharmacological research. Furthermore, we acknowledge that the absence of continuous dynamic monitoring of ALP across different time-points is a limitation of this study. Future studies must incorporate receptor antagonists across multiple dynamic time-points to definitively confirm signaling specificity and rule out the influence of endogenous adenosine. Lastly, while we utilized a hybrid model (MC3T3-E1 for osteoblast and primary BMMs for osteoclast) to optimize functional reliability for each assay, the lack of an autologous co-culture system derived from the exact same animal limits our ability to evaluate direct intercellular crosstalk. The MC3T3-E1 line was selected to ensure phenotypic stability during long-term mineralization (28 days), while primary BMMs were utilized to maximize physiological relevance in short-term differentiation assays. It is worth noting that both models share a C57BL/6 genetic background, supporting the biological consistency of the findings [39]. Future studies utilizing autologous co-culture systems derived from the same animal would further validate the intercellular crosstalk in a more physiological context.

Nonetheless, our findings establish PDRN as a selective anabolic agent that promotes osteoblast differentiation and offer a compelling hypothesis for its mechanism of action. Given its clinical use and favorable safety profile, PDRN may represent a valuable addition to current treatment options for osteoporosis and bone regeneration.

## 4. Materials and Methods

### 4.1. Reagents

Polydeoxyribonucleotide (PDRN, Newcleo^®^) was purchased from Yungjin Pharm. Co., Ltd. (Seoul, Republic of Korea). Alpha-minimum essential medium (α-MEM), fetal bovine serum (FBS), and penicillin–streptomycin were purchased from Gibco (Grand Island, NY, USA). Ascorbic acid and β-glycerophosphate were purchased from Sigma-Aldrich (St. Louis, MO, USA). Alizarin Red S was purchased from BioSolution Co., Ltd. (Seoul, Republic of Korea). Recombinant mouse M-CSF was purchased from PeproTech (Cranbury, NJ, USA). Recombinant human RANKL was purchased from R&D Systems (Minneapolis, MN, USA). The Alkaline Phosphatase (ALP) staining kit (StemTAG^TM^) was obtained from Cell Biolabs, San Diego, CA, USA. The TRAP staining solution was prepared by dissolving 0.1 mg/mL naphthol-AS-MX phosphate (Sigma-Aldrich, St. Louis, MO, USA) and 0.3 mg/mL Fast Red Violet LB salt (Sigma-Aldrich) in 0.1 M sodium acetate buffer (pH 5.0) containing 50mM sodium tartrate. N,N-dimethylformamide (0.83%; Sigma-Aldrich) was used to solubilize the naphthol substrate. For cell viability assays, CellTiter 96^®^ AQueous One Solution (MTS) was obtained from Promega (Madison, WI, USA). Primary antibodies against Runx2 (sc-390351), Osterix (sc-393325), and β-actin (sc-47778) were purchased from Santa Cruz Biotechnology (Dallas, TX, USA). All other chemicals were of analytical grade.

### 4.2. Cell Culture

To assess the effect of PDRN on osteoblastogenesis, MC3T3-E1 Subclone 10 pre-osteoblasts were kindly provided by Professor Je-Yong Choi (School of Medicine, Kyungpook National University, Daegu, Republic of Korea). This cell line was selected for its well-established osteogenic potential [40]. Unlike primary osteoblasts, which often exhibit significant donor-to-donor variability, this specific cell line offers a genetically stable and homogeneous population with high mineralization potential, ensuring the reproducibility required for mechanistic analysis [41]. To ensure phenotypic stability and experimental reproducibility, cells between passages 10 and 15 were strictly used for all experiments. They were seeded at a density of 3 × 10^4^ cells/well in a 48-well plate and cultured in osteogenic medium (α-MEM supplemented with 10% FBS, 50 μg/mL ascorbic acid, and 10 mM beta-glycerophosphate) in the presence of various concentrations of PDRN (0, 10, and 20 μg/mL). For alkaline phosphatase (ALP) staining, cells were fixed in 4% paraformaldehyde (PFA) on day 7 and stained with ALP solution (StemTAG). Stained areas were photographed, and the relative staining intensity was quantified using ImageJ software (version 1.54, NIH, Bethesda, MD, USA). Alizarin Red S (ARS) staining was performed on day 28 using ARS solution, and staining intensity was quantified using Bioquant Osteo 2019 (v19.9.60). To avoid selection bias during quantitative analysis, images were captured from four randomly selected fields per well.

For osteoclastogenesis, primary mouse bone marrow-derived macrophages (BMMs) were isolated from the femurs and tibias of 8-week-old male C57BL/6 mice, as previously described [42]. Male mice were selected to exclude the variable influence of the estrous cycle on bone marrow metabolism [43]. All animal procedures were performed in accordance with the guidelines of and were approved by the Institutional Animal Care and Use Committee (IACUC) of Kyungpook National University (KNU-2025-0412).

BMMs were freshly isolated and used immediately for experiments without serial passaging. Briefly, bone marrow cells were extracted from the long bones, and red blood cells were lysed for 2 min at room temperature. The cells were then plated in a Petri dish and cultured for 3–4 days in α-minimal essential medium (α-MEM) containing 10% fetal bovine serum (FBS) and M-CSF (30 ng/mL). This M-CSF-dependent culture system is a well-established method that selectively promotes the survival and proliferation of the monocyte/macrophage lineage, yielding a highly enriched population of BMMs while eliminating non-adherent contaminants [44]. The attached cells were lifted and used as osteoclast precursor cells (BMMs).

Subsequently, BMMs were seeded at a density of 5 × 10^3^ cells/well in 96-well plates and cultured in α-MEM containing 10% FBS, M-CSF (30 ng/mL), and RANKL (30 ng/mL). Cells were treated with PDRN at concentrations of 0, 10, and 20 μg/mL during osteoclast differentiation. For TRAP staining, cells were cultured for 4 d, fixed in 4% PFA for 1 min, refixed with ethanol/acetone (50:50) for 1 min, and stained with TRAP solution containing 0.1 mg/mL naphthol AS-MX phosphate (Sigma-Aldrich, USA), 0.3 mg/mL Fast Red Violet (Sigma-Aldrich), and 0.83% N,N-dimethylformamide (Sigma-Aldrich). For the osteoclast resorption pit assay, BMMs were cultured on autoclaved bone slices and stained on day 9. Osteoclasts were then removed, and bone slices were incubated with peroxidase-conjugated wheat germ agglutinin (Sigma-Aldrich), followed by staining with 3,3-diaminobenzidine (Sigma-Aldrich).

### 4.3. Cell Metabolic Activity Assay

Cell metabolic activity was evaluated using the 3-(4,5-dimethylthiazol-2-yl)-5-(3-carboxymethoxyphenyl)-2-(4-sulfophenyl)-2H-tetrazolium (MTS) assay. MC3T3-E1 cells were seeded in 96-well plates at a density of 5 × 10^3^ cells/well and treated with PDRN at concentrations of 0, 10, 20, 50, and 100 μg/mL in osteogenic medium. BMMs were seeded at the same density and treated with PDRN (0, 10, and 20 μg/mL) in the presence of M-CSF (30 ng/mL). After 24 h of incubation, the culture medium was replaced with 120 µL of α-MEM containing 20 µL of CellTiter 96 AQueous One Solution (Promega, Madison, WI, USA). Plates were incubated for an additional 4 h at 37 °C in a humidified atmosphere with 5% CO_2_, and absorbance was measured at 490 nm using a microplate reader.

### 4.4. Immunofluorescence

MC3T3-E1 cells were fixed with 4% PFA for 1 min, permeabilized with 0.1% Triton X-100, and blocked with 1% bovine serum albumin (BSA). Cells were incubated with primary antibodies against Runx2 (1:1000 dilution) and Osterix (1:1000 dilution) for 90 min at 25 °C, followed by three washes with PBS containing 0.1% Tween-20 (PBS-T). Rhodamine-conjugated secondary antibodies and 4′,6-diamidino-2-phenylindole (DAPI) were used for nuclear counterstaining. Fluorescence images were captured using a Leica fluorescence microscope. For quantitative analysis, images from four selected fields per well were captured. To prevent selection bias, images were acquired using a systematic sampling strategy based on predefined coordinates (four cardinal points: 3, 6, 9 and 12 o’clock positions) within each well. Furthermore, focusing was performed using the DAPI channel to ensure the investigator was blinded to the target protein fluorescence intensity during image acquisition. The fluorescence intensity of Runx2 and Osterix was quantified using ImageJ software (version 1.54, (NIH, Bethesda, MD, USA) data are presented as mean ± SD.

### 4.5. Total RNA Isolation and Gene Expression Analysis

Total RNA was extracted from MC3T3-E1 cells and osteoclasts using an easy-BLUE Total RNA Extraction Kit (iNtRON Biotechnology, Seongnam-si, Gyeonggi-do, Republic of Korea), following the manufacturer’s instructions. Complementary DNA (cDNA) was synthesised from 2 μg of total RNA using SuperScript II Reverse Transcriptase (Invitrogen, CA, USA). Quantitative real-time PCR (qRT-PCR) was performed using Power SYBR Green Master Mix (Applied Biosystems, Foster, CA, USA). Primers were designed using Primer Express (Applied Biosystems), and their sequences are listed in Table A1. Relative gene expression levels were calculated using the comparative *C*_t_ method (2^−∆∆*C*t^) and normalized to the housekeeping gene GAPDH.

### 4.6. Western Blotting Analysis

Total protein lysates were prepared from MC3T3-E1 cells using M-PER Mammalian Protein Extraction Reagent (Thermo Fisher Scientific, Rockford, IL, USA) supplemented with protease and phosphatase inhibitors.

Proteins were separated by 12% sodium dodecyl sulfate–polyacrylamide gel electrophoresis (SDS-PAGE) and transferred onto polyvinylidene difluoride (PVDF) membranes. Western blotting was performed as described previously using antibodies against Runx2 (sc-390351), Osterix (sc-393325) and β-actin (sc-47778).

### 4.7. Statistical Analysis

All quantitative data are presented as mean ± standard deviation (SD). For cell metabolic activity and cell counting assays, experiments were performed with five independent biological replicates (*n* = 5). For other molecular assays (qRT-PCR, staining), experiments were performed with at least three independent biological replicates (*n* = 3–5), as specified in the respective figure legends. Western blotting was performed as a qualitative confirmatory assay, without quantification. The normality of data distribution was assessed using the Shapiro–Wilk test. All datasets exhibited a normal distribution (*p* > 0.05), and no data points were excluded. Since data followed a normal distribution, statistical comparisons were performed using one-way analysis of variance (ANOVA) followed by Tukey’s post hoc test for multiple comparisons. A *p*-value < 0.05 was considered statistically significant.

## 5. Conclusions

PDRN exerts a selective anabolic effect by inducing growth arrest (reduced cell number) and a metabolic shift consistent with cellular differentiation, characterized by a transient modulation of metabolic activity followed by enhanced osteogenic marker expression. This effect was consistently validated at molecular (Runx2/Osterix), enzymatic (ALP), and functional (mineralization) levels. In contrast, PDRN had no direct functional impact on osteoclasts. These findings provide in vitro evidence supporting a plausible working hypothesis in which PDRN enhances bone formation through the robust upregulation of osteogenic transcription factors. While further investigations utilizing selective antagonists and multiple time-points are strictly required to definitively elucidate the precise purinergic receptor mechanisms, our phenotypic data highlight the potential of PDRN as a candidate for safe and effective therapeutic strategy for bone regeneration and metabolic bone diseases such as osteoporosis.

## Data Availability

The original data presented in the study are openly available in FigShare at https://figshare.com/s/ac514ccd5a1b75a2f8d6 (accessed on 25 February 2026).

## Supplementary Materials

The following supporting information can be downloaded at https://www.mdpi.com/article/10.3390/md24030100/s1, Figure S1: Time-course analysis of MC3T3-E1 metabolic activity.

## Abbreviations

The following abbreviations are used in this manuscript:

*α*-MEM	Alpha-minimum essential medium
ADP	Adenosine diphosphate
ALP	Alkaline phosphatase
ANOVA	Analysis of variance
ARS	Alizarin Red S
ATP	Adenosine triphosphate
BMMs	Bone marrow-derived macrophages
BSA	Bovine serum albumin
cDNA	Complementary DNA
CTSK	Cathepsin K
DAPI	4′,6-diamidino-2-phenylindole
FBS	Fetal bovine serum
GAPDH	Glyceraldehyde 3-phosphate dehydrogenase
GPCRs	G-protein-coupled receptors
IACUC	Institutional Animal Care and Use Committee
M-CSF	Macrophage colony-stimulating factor
MTS	3-(4,5-dimethylthiazol-2-yl)-5-(3-carboxymethoxyphenyl)-2-(4-sulfophenyl)-2H-tetrazolium
NFATc1	Nuclear factor of activated T-cells, cytoplasmic 1
PBS	Phosphate-buffered saline
PDRN	Polydeoxyribonucleotide
PFA	Paraformaldehyde
PTH	Parathyroid hormone
PVDF	Polyvinylidene difluoride
qRT-PCR	Quantitative real-time polymerase chain reaction
RANKL	Receptor activator of nuclear factor kappa-B ligand
SD	Standard deviation
SDS-PAGE	Sodium dodecyl sulfate–polyacrylamide gel electrophoresis
TRAP	Tartrate-resistant acid phosphatase

## Appendix A

**Table A1:** Primer sequences for qRT-PCR analysis of osteogenic and osteoclastic genes.

Targets	Forward (5′→3′)	Reverse (5′→3′)
Runx2 (mouse)	ACA TGG CCA GAT TCA CAG TGG	TGG TGC CCG TTA GCA ATT G
Osterix (mouse)	ATG CTC CGA CCT CCT CAA CTT T	GGA AAA CGG CAA ATA GGA TTG G
Osteocalcin (mouse)	TTC TGC TCA CTC TGC TGA CCC T	CCT GCT TGG ACA TGA AGG CTT
Cathepsin K (mouse)	TGC GGC ATT ACC AAC ATG G	TCC AAA GCC ACC AAT ATC TTG C
TRAP (mouse)	TCC CCA ATG CCC CAT TC	CGG TTC TGG CGA TCT CTT TG
NFATc1 (mouse)	GAT CCC GTT GCT TCC AGA AAA T	TCT GTC TCC CCT TTC CTC AGC T
GAPDH (mouse)	GCA TCT CCC TCA CAA TTT CCA	GTG CAG CGA ACT TTA TTG ATG G
