# Supplementary material for: Polydeoxyribonucleotide (PDRN) Selectively Promotes Osteoblast Differentiation Without Affecting Osteoclastogenesis
Source: Mar Drugs. 2026 Mar 3;24(3):100. doi: 10.3390/md24030100 (PMC13028593; doi:10.3390/md24030100)
Supplement: Supplementary file 1 [file marinedrugs-24-00100-s001.zip › FigureS1_caption.pdf]

Figure S1. Time-course analysis of MC3T3-E1 metabolic activity. To assess cell metabolic activity over time and exclude potential cytotoxicity, an MTS assay was performed with increased biological replicates (N = 10). MC3T3-E1 cells were treated with PDRN (0, 10, and 20 μg/mL) for 24 and 48 h. While a transient reduction in metabolic activity was observed at 24 h in the PDRN-treated groups (**$p < 0.01$), metabolic activity significantly increased in all groups at 48 h compared to the 24 h time point. This recovery indicates that the cells remained viable and maintained growth potential. Data represent the mean ± SD of ten independent biological replicates. Statistical significance was determined by one-way ANOVA followed by Tukey's post-hoc test. **$p < 0.01$ vs. Control.
