# Supplementary figures and images for: Polydeoxyribonucleotide (PDRN) Selectively Promotes Osteoblast Differentiation Without Affecting Osteoclastogenesis
Source: Mar Drugs. 2026 Mar 3;24(3):100. doi: 10.3390/md24030100 (PMC13028593; doi:10.3390/md24030100)

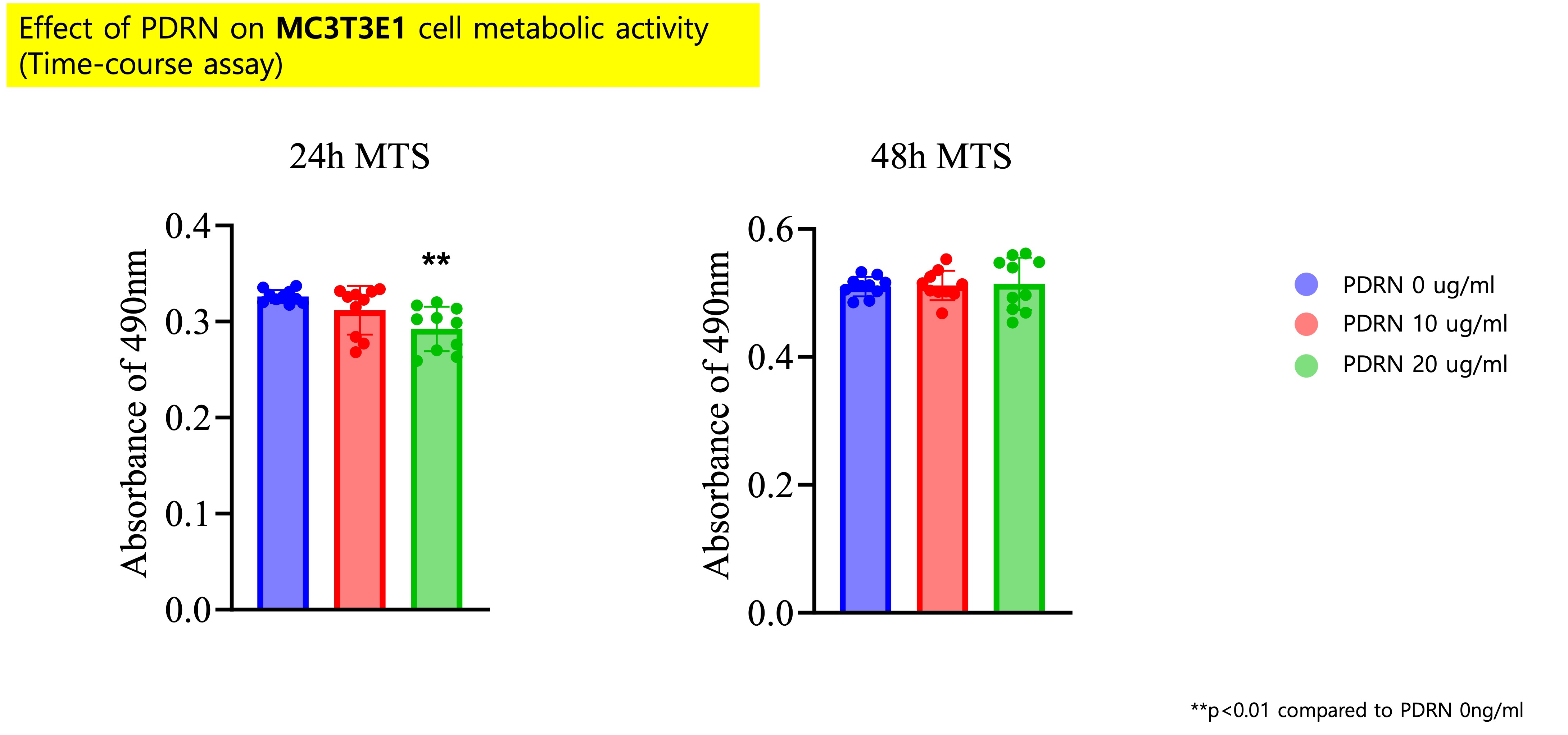

Supplement: Supplementary file 1 [file marinedrugs-24-00100-s001.zip › figureS1.jpg]
